# Case report: First case of neuromelioidosis in Europe: CNS infection caused by *Burkholderia pseudomallei*

**DOI:** 10.3389/fneur.2022.899396

**Published:** 2022-07-29

**Authors:** Nikolaos G. Dimitriou, Greta Flüh, Sabine Zange, Aykut Aytulun, Bernd Turowski, Hans-Peter Hartung, Sven G. Meuth, Michael Gliem

**Affiliations:** ^1^Department of Neurology, Medical Faculty, Heinrich-Heine-University, Duesseldorf, Germany; ^2^Labor Dr. Wisplinghoff, Cologne, Germany; ^3^Bundeswehr Institute of Microbiology, Munich, Germany; ^4^Department of Diagnostic and Interventional Radiology, Medical Faculty, Heinrich-Heine-University, Duesseldorf, Germany

**Keywords:** neuromelioidosis, Europe, case report, MRI, *Burkholderia pseudomallei*

## Abstract

Neuromelioidosis is a rare CNS infection caused by *Burkholderia pseudomallei* and is characterized by high morbidity and mortality. Our report presents the diagnostic and therapeutic approach of the first case of neuromelioidosis confirmed in Europe. A 47-year-old man with a medical history of recurrent otitis with otorrhea and fever after tympanoplasty and radical cavity revision operation on the left ear was admitted with headache, decreased level of consciousness, dysarthria, left-sided hemiparesis, and urinary incontinence. After extensive investigations including MRI, microbiological, serological, and CSF analyses, and, ultimately, brain biopsy, a diagnosis of neuromelioidosis was established. Despite antibiotic treatment, the patient showed no clinical improvement and remained in a severely compromised neurological state under mandatory mechanical ventilation. Neuromelioidosis can pose a diagnostic challenge requiring an extensive diagnostic evaluation because of its uncommon clinical and radiological presentations.

## Introduction

Melioidosis is a rare infectious disease caused by *Burkholderia pseudomallei*, a Gram-negative, aerobic, and non-spore-forming, soil saprophyte. The disease is endemic to tropical regions such as northern Australia and Southeast Asia (1). According to growing evidence, melioidosis may also be endemic in the Indian subcontinent and the Caribbean (2, 3). Typical manifestations of the disease include abscess formation especially in the lungs, liver, spleen, skin, and skeletal muscle. The involvement of the central nervous system is rare (1–5%) but statistically associated with higher case mortality up to 25% (4, 5).

Here, we present the first case of neuromelioidosis confirmed in Europe based on detection of the bacterium in cerebrospinal fluid, brain bioptic sample, and smear of the left ear.

## Case presentation

A 47-year-old man presented to an external department of otolaryngology with deterioration of general condition and fever. The diagnostic investigation did not identify any infectious source. Regarding the medical history of the patient, antibiotic treatment had been repeatedly administered because of recurrent otitis with otorrhea and fever after tympanoplasty and radical cavity revision operation on the left ear approximately 6 months before the current presentation. The patient works as exhibition organizer and therefore often travels internationally. However, in the preceding 2 years, he did not travel outside Europe and was mainly located in Germany or Italy according to his family. Two days after hospital discharge, the patient presented again in the emergency department of the same clinic with headache, decreased level of consciousness, dysarthria, left-sided hemiparesis, and urinary incontinence. The brain CT-scan was normal. One week after admission, the patient was transferred to our hospital for the purpose of a neurological assessment.

Upon arrival, the patient showed decreased level of consciousness, left-sided hemiparesis, and urinary incontinence. The lumbar puncture revealed signs compatible with CNS infection (glucose 19 mg/dl, lactate 3.9 mmol/l, protein 206 mg/dl, and leukocytes 103/μl), whereupon treatment with empiric intravenous triple therapy with acyclovir, ampicillin, and ceftriaxone was initiated. The new CT scan of the brain revealed partial compression of the right lateral ventricle. Subsequently, the patient was admitted to our general neurology ward for further diagnostic investigation. The Brain MRI showed FLAIR-hyperintense bihemispheric lesions (significantly more on the right than on the left) in the area of the thalamus and the internal capsule, and extensive brainstem involvement with faint contrast enhancement in the posterior limbs of the internal capsule along with the parapontine region. The progressive deterioration of the patient's level of consciousness resulted in transfer to our intermediate care unit and subsequently to our intensive care unit because of further worsening with a score of 6 on the Glasgow Coma Scale. Upon admission to the intensive care unit, the patient was hemodynamically stable, free of vasopressor support, and received 2 L of oxygen per minute *via* nasal cannula. Protective endotracheal intubation was performed within a short timeframe as a result of the prolonged comatose state with limited protective brainstem reflexes. Because the X-ray confirmed bilateral pneumonia, presumably triggered by microaspiration, the antibiotic regimen was switched to piperacillin/tazobactam and clarithromycin.

An extensive differential diagnostic, including cerebrospinal fluid (CSF) analysis, was carried out with regard to encephalitis of infectious, autoimmune, or malignant etiology. PCR testing for HSV 1/2, VZV, JCV, and HHV-6 was negative, and no organisms were isolated by culture. Anti-Zic4 antibodies were weakly positive in the CSF. A tumor search, including a chest and abdomen CT scan, showed no evidence of neoplasy. A smear culture of the drainage deriving from the left ear isolated *B. pseudomallei*. The supplementary imaging of the mastoid and the otolaryngological examination did not provide any evidence of mastoiditis or abscess. However, the antibiotic regimen was switched to ceftazidime in accordance with the antibiogram. The follow-up MRI imaging of the brain on day 4 showed an increasing size of the cerebral lesions and radiological features of elevated intracranial pressure. Consequently, an external ventricular drain system (EVD) was placed for intracranial pressure monitoring and management. For diagnostic clarification, stereotactic brain biopsy was performed. The histopathological assessment revealed an inflammatory process that could not be further specified. With a provisional diagnosis of an autoimmune/inflammatory encephalitis, intravenous methylprednisolone pulse treatment was initiated. In the further course, plasma separation was also initiated because of suspicion of an autoimmune inflammatory etiology. However, from both the CSF and brain biopsy samples, which were sent for a supplementary examination to the Bundeswehr Institute of Microbiology in Munich, *B. pseudomallei* was isolated. Based on the radiological and microbiological findings as well as the clinical presentation, which strongly indicated the presence of neuromelioidosis, the antibiotic treatment was escalated to meropenem. The re-evaluation of the mastoid CT-imaging revealed an exposed bone defect in the mastoid cavity of the left ear, which might have been the entry point of the detected pathogen to the CNS. Therefore, an uncomplicated radical cavity revision with bone reconstruction was carried out in collaboration with our otorhinolaryngology department. Another follow-up MRI imaging of the brain on day 25 revealed regression of the inflammatory changes but with significant residual florid inflammatory supra- and infratentorial lesions. Due to stable intracranial pressure in the range of normal standards, the EVD could be removed and the dexamethasone treatment gradually tapered. Nevertheless, no improvement in the neurological condition could be observed during the entire in-patient stay. In the further course, tracheostomy was performed as a result of the prolonged comatose state with mandatory mechanical ventilation due to lack of spontaneous breathing. Furthermore, a port system was placed for further administration of meropenem (6 weeks in total) followed by a maintenance phase with oral antibiotic treatment with cotrimoxazole for 6 months. At discharge, the patient showed no further clinical improvement and remained in the comatose state under mandatory mechanical ventilation. The patient was transported to a post-acute care facility for the purpose of further antibiotic administration.

The differential diagnosis for this presentation was wide. It included infectious, primary autoimmune, and malignant etiologies. The group of infectious causes included central nervous system (CNS) toxoplasmosis, tuberculous encephalitis, viral meningoencephalitis, listeriosis, and brucellosis. None of these organisms were isolated. Malignant differential diagnoses included CNS lymphoma. No malignant cells were identified on CSF cytology and cerebral biopsy.

## Discussion

**Figure 1:**
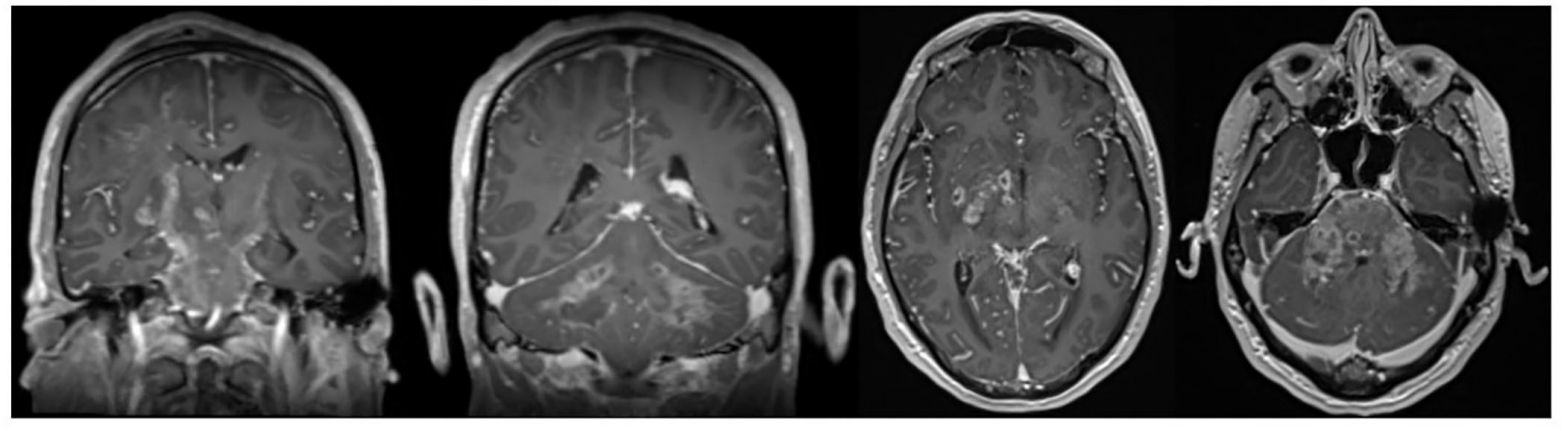
Axial and coronal contrast-enhanced T1-weighted images showing typical rim-enhancing microabscesses spreading along white matter tracts.

Neuromelioidosis is a rare infection of the nervous system caused by *Burkholderia pseudomallei*. It accounts for approximately 3% of all melioidosis cases and is statistically associated with much higher morbidity and mortality rate. The transmission of melioidosis is by direct inoculation, inhalation, or ingestion (6). Our case demonstrates an additional mechanism of accessing the CNS *via* possible entry of the detected pathogen through an exposed mastoid bone defect. Extensive involvement of the brainstem in MRI imaging is common. This could be observed in our case, whose brain-MRI revealed bihemispheric distinctly right-sided accentuated FLAIR hyperintensities as well as rim-enhancing microabscesses spreading along white matter tracts predominantly in corticospinal tracts and cerebral peduncles (Figure 1). Bearing this typical pattern in mind, the rare disease might get diagnosed more often, and initiating therapy in early phases might save patients from persistent disability and death. However, whether the initial immunosuppressant therapy in our case was beneficial by limiting CNS immunoreaction or detrimental by weakening the antimicrobial response remains speculative. An additional frequent radiological feature of neuromelioidosis is thickening of trigeminal nerves, which could also be observed in the brain MRI scans of our patient. This radiological finding may contribute to the identification of the pathogen's entry mechanisms into the CNS potentially through direct axonal transport in cranial nerves (7). Our patient provided a diagnostic predicament as the initial clinical presentation, MRI and, negative cultures delayed the identification of the pathogen and thus the early initiation of appropriate antibiotic treatment. We present the first case of neuromelioidosis in Europe with a severe neurological outcome.

## Conclusions

Neuromelioidosis presentation can mimic other inflammatory or infectious conditions. Bearing the typical MRI pattern with typical rim-enhancing microabscesses spreading along white mater tracts in mind, this rare disease might get diagnosed more often, and initiating therapy in early phases might save patients from persistent disability and death.

## Data availability statement

The original contributions presented in the study are included in the article/supplementary material, further inquiries can be directed to the corresponding author.

## Ethics statement

Ethical review and approval was not required for the study on human participants in accordance with the local legislation and institutional requirements. The patients/participants provided their written informed consent to participate in this study.

## Author contributions

ND and MG: acquisition of the data and drafting of the manuscript. GF, SZ, and BT: acquisition and interpretation of the data. AA: acquisition of the data and revision of the manuscript. SGM and H-PH: revision of the manuscript for intellectual content. All authors contributed to the article and approved the submitted version.

## Conflict of interest

The authors declare that the research was conducted in the absence of any commercial or financial relationships that could be construed as a potential conflict of interest.

## Publisher's note

All claims expressed in this article are solely those of the authors and do not necessarily represent those of their affiliated organizations, or those of the publisher, the editors and the reviewers. Any product that may be evaluated in this article, or claim that may be made by its manufacturer, is not guaranteed or endorsed by the publisher.

## Abbreviations

CNS: central nervous system
CSF: cerebrospinal fluid
CT: computer tomography
EVD: external ventricular drain
FLAIR: fluid-attenuated inversion recovery
HHV-6: human herpesvirus 6
HIV: human immunodeficiency virus
HSV-1/HSV-2: herpes simplex virus type 1/type 2
IV: intravenous
JCV: John Cunningham virus
MRI: magnet resonance imaging
PCR: polymerase chain reaction and
VZV: varicella zoster virus.
